# Objective Analysis of the Impact of Facial Neuromuscular Electrical Stimulation on Facial Muscle Morphology

**DOI:** 10.1093/asjof/ojaf154

**Published:** 2026-01-14

**Authors:** Itsuko Okuda, Mizuho Takeda, Kiyoko Kato, Naoki Yoshioka

## Abstract

**Background:**

Neuromuscular electrical stimulation (NMES) has gained popularity as a noninvasive approach for facial rejuvenation. Although NMES is known to increase muscle mass, its effects on facial muscle morphology and the relationship between muscle changes and the overlying facial contour have not been elucidated.

**Objectives:**

To evaluate the effects of facial NMES on muscle mass and facial contours using 3-dimensional computed tomography (CT).

**Methods:**

Fifteen healthy women were enrolled in this prospective split-face trial. One participant was excluded because of noncompliance, and data from 14 participants were analyzed. The NMES was applied to the left infraorbital and temple areas using a periorbital-specific flexible thin-film electrode device, with the right side serving as the control. Computed tomography examinations were conducted at baseline and after 8 weeks of NMES application. The thicknesses of the orbicularis oculi muscle (OOM) and temporal muscle, and the length of orbital fat herniation and temporal contours were quantitatively assessed.

**Results:**

On the NMES-applied side, OOM thickness significantly increased and the length of fat herniation decreased (*P* < .001). Furthermore, temporal muscle thickness increased and the temple contour improved significantly on the NMES-applied side compared with the control side (*P* < .001). Strong negative correlations were observed between muscle thickness and the corresponding morphological changes (OOM vs length of fat herniation, *r* = −0.73; temporal muscle vs temple contour, *r* = −0.71).

**Conclusions:**

In this pilot study, facial NMES enhanced muscle mass and ameliorated age-related morphological changes, suggesting its potential as a noninvasive strategy for facial rejuvenation.

**Level of Evidence: 3 (Therapeutic):**

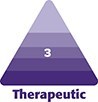

Interest in antiaging therapies and aesthetic procedures has steadily increased in recent years. Subsequently, neuromuscular electrical stimulation (NMES), which induces muscle contractions by passively stimulating peripheral nerves through external electrical impulses,^1-3^ has increasingly attracted attention. It is widely used to enhance muscle mass and strength with a minimal physical load within a relatively short duration. Furthermore, NMES is applied to facial regions to address signs of ageing.^4-8^

A flexible thin-film facial NMES electrode device, specifically designed with hydrogel patches to fit the infraorbital to the temple areas, was developed to target age-related morphological changes around the eyes (Video). However, previous evaluations of the effects of NMES on the face have been largely limited to superficial appearance changes.^4,8^ To date, the impact of NMES on the underlying facial muscle mass and the relationship between muscle changes and overlying facial contour alterations have not been elucidated.

Recent advancements in multidetector-row computed tomography (MDCT) and image analysis technology have facilitated the generation of highly detailed 3-dimensional (3-D) CT images.^7,8^ Multidetector-row computed tomography provides comprehensive anatomical information and allows 3-D reconstruction of both external surfaces and internal structures. Additionally, these CT images enable a detailed understanding of the anatomical relationships between the facial surface and underlying subcutaneous structures, as well as subtle anatomical changes of those structures.^9-11^

Although no consensus exists regarding the minimal clinically important difference (MCID) for improvements in orbital fat herniation or temporal hollowing, subtle changes in these regions can lead to perceptible differences in facial aesthetics. Shay et al^12^ emphasized that even small volumetric changes in the temple region can considerably impact facial appearance and patient satisfaction. Therefore, we hypothesized that quantitative changes in muscle thickness and overlying contour observed via 3-D CT could reflect clinically meaningful improvements, particularly when supported by strong anatomical correlations.

This study aimed to analyze the effects of a periorbital-specific flexible thin-film NMES electrode device equipped with hydrogel patches on the muscular structure and morphology of the face. We quantitatively evaluated changes in muscle mass and corresponding alterations in infraorbital and temple contours using high-resolution 3-D CT imaging.

## METHODS

### Study Design

This prospective study was conducted between May 2023 and July 2023 and was approved by the Institutional Review Board of Pharmaceutical Law Wisdoms (approval no. 12000111). Written informed consent was obtained from all the participants. Fifteen healthy adult female volunteers without any lesions affecting facial structures or ocular diseases were enrolled in the study. The NMES was performed in a split-face trial. An NMES device was placed on the left half of the face. No NMES device was used on the other side (control side).

### Participants

Participants were recruited from the general population through a subject recruitment agency and were selected based on their ability to voluntarily provide informed consent and their status as healthy adult women aged 40 years and older. Exclusion criteria included pregnancy or potential pregnancy, current outpatient treatment for any illness, history of facial cosmetic surgeries, conditions affecting the face because of facial nerve paralysis, trauma, or noncompliance with the study procedures.

**Figure 1.**
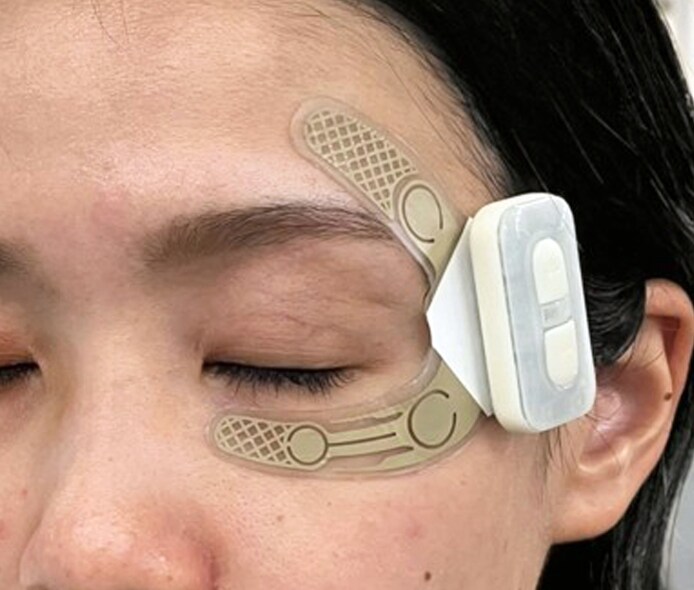
A 25-year-old female model (not a study participant) demonstrating the correct placement of the flexible thin-film neuromuscular electrical stimulation electrode device. The electrode was applied from the infraorbital region to the temple area on the left side of the face, followed by attachment of the main stimulation unit. Each session lasted 10 minutes.

All participants received standardized instructions regarding the appropriate use of a periorbital-specific flexible thin-film NMES electrode device (22G1XX; YA-MAN Ltd, Tokyo, Japan) and were instructed to apply the device from the left lower eyelid to the temple (Figure 1). To minimize confounding variables, participants were instructed to maintain their usual skincare regimen from 4 weeks before study initiation until study completion and to abstain from undergoing any aesthetic treatments or cosmetic medical procedures during the study period. Additionally, participants were advised to refrain from consuming dietary supplements throughout the study period.

The use of the NMES device commenced after the baseline facial CT scan. The participants used the device 3 times per week, predominantly in the evening for 10 minutes per session. The sessions were conducted on nonconsecutive days (eg, Sunday, Tuesday, and Thursday), and consecutive-day usage (eg, Sunday through Tuesday) was avoided to ensure adequate muscle recovery. A follow-up facial CT scan was performed after 8 weeks of consistent use.

### Image Acquisition

For all CT examinations, we used a 192 × 2 dual-source CT scanner (Somatom Force; Siemens Healthcare, Forchheim, Germany) with the following parameters: tube voltage, 120 kVp; tube current, 150 mA; exposure time, 1.0 second; and slice thickness, 0.5 mm. Whole-face scans were obtained for each participant in the supine and coronal facial positions.

All CT imaging data were transferred to a workstation (ZioCube; Ziosoft Inc., Tokyo, Japan), where 3-D CT images and reconstructed images were generated. The workstation is equipped with multiple software applications designed to create 3-D CT images and reconstruct CT image representations of the face.

### Analysis of CT Images

Computed tomography images were jointly analyzed by two radiologists, each with more than 25 years of experience. Each measurement item of the infraorbital and temporal regions was measured 3 times using a digital caliper available on the workstation, and the average values were calculated.

#### Assessment of the Attachment Site of a Neuromuscular Electrical Stimulation Electrode Device

**Figure 2.**
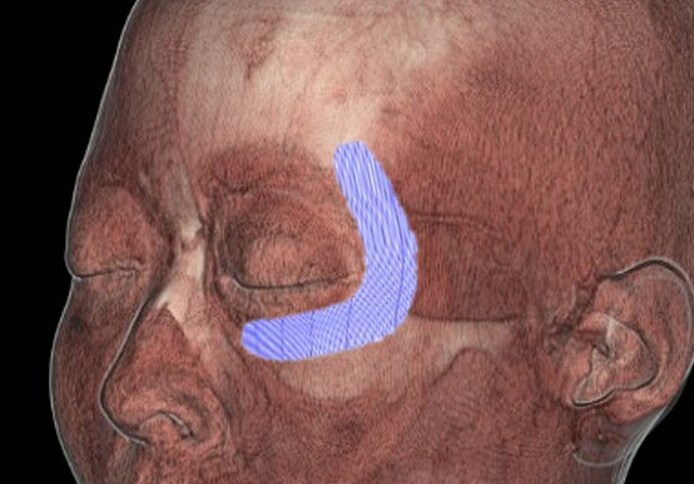
Three-dimensional computed tomography (CT) image of the facial expression muscles overlaid with a semi-transparent white skin layer. For illustrative purposes, a digitally superimposed rendering of the flexible thin-film neuromuscular electrical stimulation (NMES) electrode (purple mesh) was placed on the left side of the face to conceptually demonstrate the spatial relationship of the NMES electrode with the underlying orbicularis oculi and temporal muscles. CT imaging was not used to confirm device placement in each participant during actual use.

The initial step involved the identification and standardization of the anatomical regions to be analyzed for NMES-induced changes. Accordingly, 3-D CT images were generated to approximate the anatomical relationship between the intended NMES device placement sites and underlying subcutaneous structures. The electrode overlay in the reconstructed images was based on the standardized application protocol rather than on the actual device placement during treatment sessions. This approximation was used to assess the typical spatial relationship of the device with the orbicularis oculi muscle (OOM) and temporal muscles (Figure 2).

#### Measurement of the Infraorbital Area

**Figure 3.**
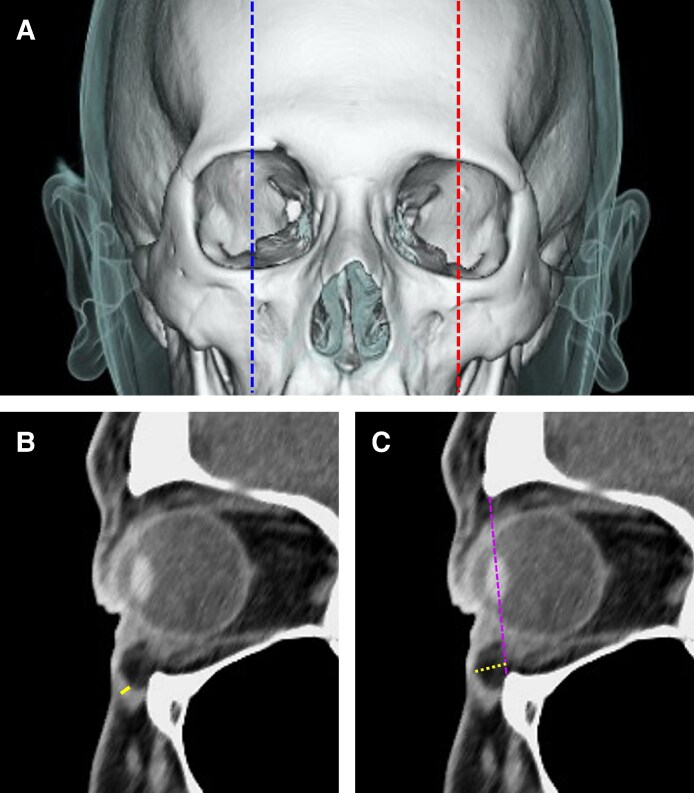
(A) Three-dimensional computed tomography (CT) image of the facial bone overlaid with a semi-transparent green skin layer. To assess orbicularis oculi muscle (OOM) thickness and the length of orbital fat herniation, sagittal CT images through the orbital center were reconstructed along the dashed lines for both the control (left: blue dashed lines) and neuromuscular electrical stimulation-applied (right: red dashed lines) sides. (B) Reconstructed sagittal CT image. OOM thickness is defined as the distance between the anterior and posterior surfaces of the muscle, indicated by the yellow line. (C) Another reconstructed sagittal CT image. The purple dashed lines running from the anterior edge of the orbital roof to the anterior edge of the orbital floor was used as a reference for measuring orbital fat prolapse length. Orbital fat prolapse length is defined as the length of fat herniation (measured as the length of the straight orange line).

Sagittal CT images through the orbital center were reconstructed along the dotted line on both the right (control) and left sides (NMES-applied) to assess OOM thickness and the length of orbital fat herniation (Figure 3). Orbicularis oculi muscle thickness was defined as the distance from the anterior to the posterior surface of the OOM. The length of orbital fat herniation was defined as the perpendicular distance from a reference line connecting the anterior edges of the orbital roof and floor to the posterior surface of the OOM.

These measurements were obtained for all the participants before and after the intervention. The change rates of OOM thickness and the length of orbital fat herniation were calculated using the following formula:

$$\begin{array}{rl} \mathrm{Change}\;\mathrm{rate}\;(\text{\%})\;= & \left(\;\mathrm{post}\text{-}\mathrm{interventionvalue}\text{–}\mathrm{pre}\text{-}\mathrm{interventionvalue}\right)\\ & /\mathrm{pre}\text{-}\mathrm{interventionvalue}\;\times \;100. \end{array}$$

#### Measurement of the Temple Area

**Figure 4.**
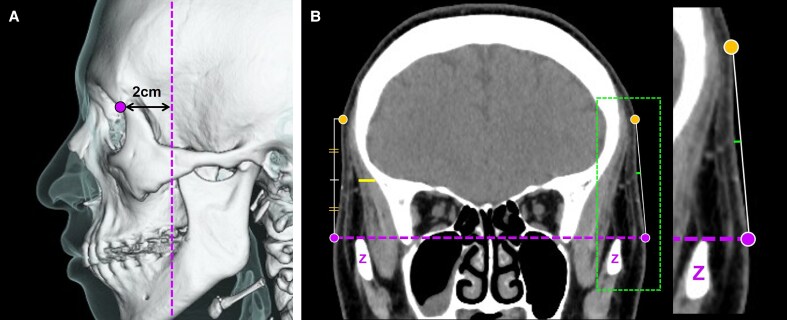
(A) To evaluate the thickness of the temporal muscle and the degree of the temple contour, coronal computed tomography (CT) images were reconstructed 2 cm dorsal to the lateral surface (indicated by a purple circle) of the superior orbital rim, along the purple dashed lines. Measurements were obtained on both the control and the neuromuscular electrical stimulation-applied sides. (B) The thickness of the temporal muscle (length of the yellow line) was measured at the level of the midpoint of a line connecting the surface of the maximal head circumference (indicated by a yellow circle) and the superior margin of the zygomatic arch (indicated by purple circles and dashed lines). In the same coronal CT image (magnified in the right panel), a reference line was drawn connecting the surface of the maximal head circumference and the superior margin of the zygomatic arch. The perpendicular distance (green line) from this reference line to the surface of the temple area was defined as the “degree of the temple contour”.

Coronal CT images were reconstructed 2 cm dorsal to the lateral surface of the superior orbital rim to evaluate the thickness of the temporal muscle and the degree of temple contour (Figure 4). Temporal muscle thickness was measured at the midpoint of the line connecting the surface of the maximal head circumference and the superior margin of the zygomatic arch.

A reference line was drawn on the same coronal image connecting the surface of the maximal head circumference and the superior margin of the zygomatic arch. The perpendicular distance from this reference line to the surface of the temple area was defined as the degree of temple contour, which was used to assess the morphological changes in the temple area.

These measurements were conducted for all participants on both the control and NMES-applied sides before and after the intervention. The change rates in the temporal muscle thickness and temple contour were calculated as follows: change rate (%) = (post-intervention value − pre-intervention value)/pre-intervention value × 100.

A positive change rate in the temple area was defined as a reduction in temporal hollowing or an enhancement of temporal protrusion. Conversely, worsening temporal hollowing or a reduction in temporal protrusion was expressed as a negative change rate.

### Subsidiary Investigation

To assess subjective satisfaction, a product-use questionnaire was administered at weeks 4 and 8 following NMES initiation. Participants were asked to evaluate both the NMES-applied and control sides using a 5-point Likert scale, comprising the following response options: “very dissatisfied,” “dissatisfied,” “neutral,” “satisfied,” and “very satisfied.” The questionnaire was administered in a paper format and was not anonymous (ie, the name of each participant was included in the completed survey).

### Statistical Analysis

The ages of the participants, thickness of the OOM and temporal muscle, length of orbital fat herniation, and degree of the temple contour are presented as mean ± standard deviation. Mean pre- and post-intervention values were compared using a paired *t*-test. Moreover, the change rates between the control and NMES-applied sides were compared using an independent *t*-test to determine statistical significance. Correlation analysis was performed to determine the relationship between the rate of change in OOM thickness and the length of orbital fat herniation due to NMES and to determine the rate of change between temporal muscle thickness and temple contour. To evaluate differences in subjective satisfaction between the NMES-applied and control sides, responses from the 5-point Likert scale questionnaire were analyzed using the Wilcoxon signed-rank test.

Statistical analyses were performed using the StatMate V statistical software package (Nihon 3 B Scientific Inc., Niigata, Japan). Statistical significance was set at *P* < .05. As this was an exploratory pilot study using high-resolution 3-D CT to quantify facial morphological changes induced by NMES, no formal a priori sample size calculation was performed. Instead, a sample size of 15 participants was pragmatically selected based on resource availability, feasibility of image acquisition and analysis, and precedent from similar pilot studies in aesthetic medicine. A post hoc power analysis conducted using the observed effect sizes confirmed that the study had sufficient power (>0.80 at α = .05) to detect statistically significant changes in key outcome measures, including muscle thickness and facial contour.

## RESULTS

### Participants

Fifteen healthy adult female volunteers were enrolled in the study. One participant was excluded before the intervention started because of noncompliance with study procedures. Specifically, the individual arrived significantly late for the baseline assessment and was unable to follow instructions during image acquisition due to restlessness. As this compromised the reliability of the baseline measurements, the participant was excluded from analysis.

The remaining 14 participants completed the study. The mean age of these participants was 48.9 ± 4.9 years (range, 40-55 years). The follow-up period for each participant, including consent acquisition, the NMES application period, and post-intervention outcome assessment, was 3 months. No adverse events or complications, including ocular, dermatological, or neuromuscular issues, were observed in any participant throughout the study period.

### Analysis of CT Images

The overall 3-D CT images were of high quality and did not hinder the measurement of the state of the OOMs and temporal muscles, length of orbital fat herniation, and contours of the temple areas. In all participants, 3-D CT images confirmed precise localization of the OOM and temporal muscles directly beneath the NMES electrode device.

### Infraorbital Area

**Figure 5.**
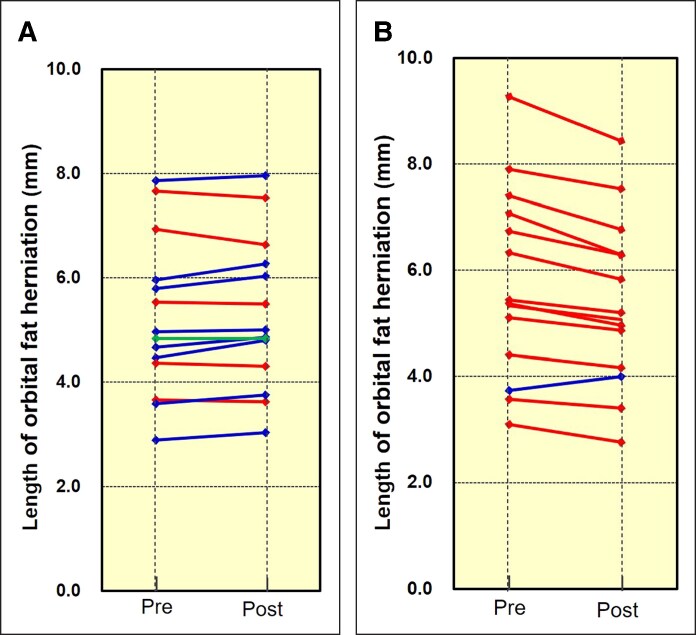
(A) Control side. Among the 14 infraorbital areas assessed, the mean value of the length of orbital fat herniation showed no substantial change (pre-intervention, 5.77 ± 1.72 mm; post-intervention, 5.40 ± 1.52 mm). Individual patterns included reduced orbital fat herniation in 4 participants (red lines), no change in 1 participant (green line), and worsening in 8 participants (dark blue lines). The mean percentage change in fat herniation was 1.63% ± 3.25%. (B) Neuromuscular electrical stimulation (NMES)-applied side. After NMES application on the 14 infraorbital areas, the mean value improved from 5.77 ± 1.72 to 5.40 ± 1.52 mm. Patterns of improvement comprised reduced fat herniation in 13 participants (red lines) and worsening in 1 participant (dark blue lines). The mean percentage change in fat herniation was −5.88% ± 4.23%, indicating a significant improvement compared with the control side.

On the control side, the mean OOM thickness was 1.47 ± 0.24 mm pre-intervention and 1.48 ± 0.24 mm post-intervention. The mean length of orbital fat herniation was 5.23 ± 1.44 mm pre-intervention and 5.30 ± 1.40 mm post-intervention. The mean change rates were 0.31% ± 1.89% for OOM thickness and 1.63% ± 3.25% for orbital fat herniation. The changes in length of fat herniation included a reduction in 5 participants, no change in 1 participant, and worsening in 8 participants (Figure 5A).

On the NMES-applied side, the mean OOM thickness was 1.31 ± 0.26 mm pre-intervention, increasing to 1.48 ± 0.25 mm post-intervention. The mean length of orbital fat herniation was 5.77 ± 1.72 mm pre-intervention, decreasing to 5.40 ± 1.52 mm post-intervention. The mean change rates were 13.74% ± 9.24% for OOM thickness and –5.88% ± 4.23% for orbital fat herniation. The changes in the length of orbital fat herniation included a reduction in 13 participants and worsening in 1 participant (Figure 5B).

**Figure 6.**
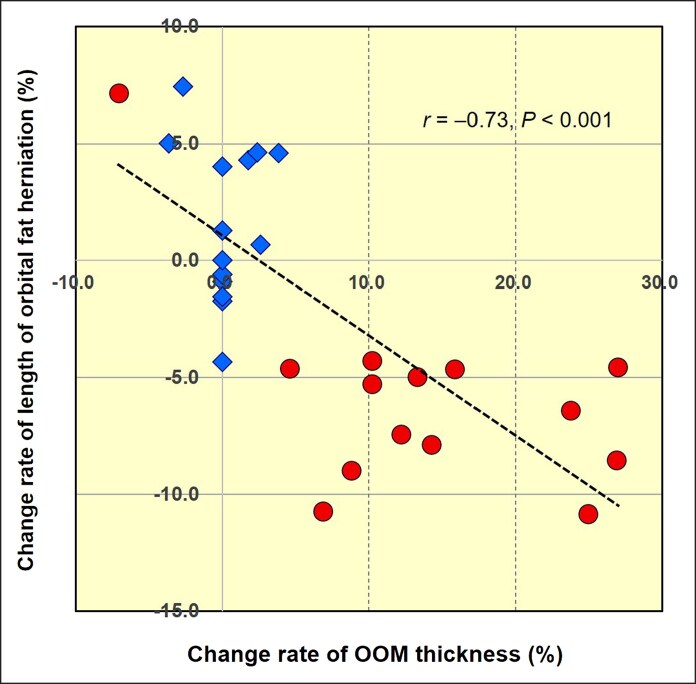
The scatter plot presents data across 28 infraorbital areas in 14 participants. Blue diamonds represent the control side, and red circles represent the neuromuscular electrical stimulation (NMES)-applied side. A significant and strong negative correlation was observed between the change rate of orbicularis oculi muscle (OOM) thickness and the change rate of the length of orbital fat herniation (*r* = −0.73, *P* < .001). These findings suggest that NMES application may lead to an increase in OOM thickness, accompanied by a corresponding reduction in the length of orbital fat herniation.

Comparative analysis revealed statistically significant differences in the change rates of both OOM thickness and the length of orbital fat herniation between the control and NMES-applied sides (*P* < .001 for both). Moreover, a strong and significant negative correlation was observed between OOM thickness and the length of orbital fat herniation across the 28 infraorbital areas assessed in 14 participants (*r* = −0.73, *P* < .001; Figure 6).

### Temple Area

**Figure 7.**
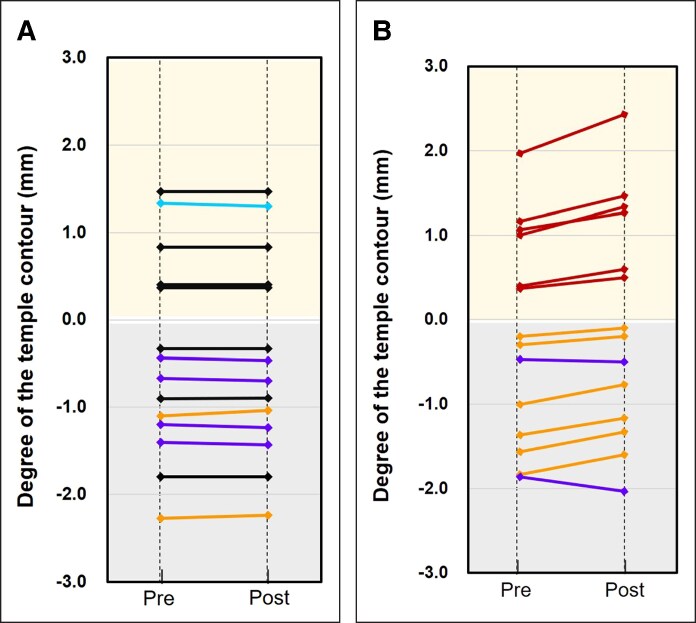
(A) Control side. Among the 14 temple areas assessed, 9 had hollowing (gray area) and 5 had protrusion (yellow area). The mean value showed no substantial change (pre-intervention, −0.41 ± 1.11 mm; post-intervention, −0.41 ± 1.10 mm). Individual patterns included reduced hollowing in 2 participants (orange lines), no change in 7 participants (black lines), exacerbated hollowing in 4 participants (purple lines), and reduced protrusion in 1 participant (light blue line). The mean percentage change in contour was −0.92% ± 3.06%. (B) Neuromuscular electrical stimulation (NMES)-applied side. Of the 14 temple areas evaluated, 8 exhibited hollowing (gray area) and 6 exhibited protrusion (yellow area). After NMES application, the mean value improved from −0.19 ± 1.19 to −0.03 ± 1.24 mm. Patterns of improvement comprised reduced hollowing in 6 participants (orange lines), enhanced protrusion in 6 participants (dark red lines), and worsened hollowing in 2 participants (purple lines). The mean percentage change in contour was 21.82% ± 17.4%, indicating a significant improvement.

On the control side, the mean temporal muscle thickness was 4.76 ± 1.58 mm pre-intervention and 4.77 ± 1.57 mm post-intervention. The degree of temple contour was 0.41 ± 1.11 mm pre-intervention and 0.41 ± 1.10 mm post-intervention. The mean change rates were 0.24% ± 0.70% for temporal muscle thickness and 0.89% ± 3.07% for temple contour. Before the intervention, the temple contour exhibited depression in 9 participants and protrusion in 5 participants. After the intervention, temple contour changes included a reduction in hollowing in 2 participants, no change in 7 participants, worsening of hollowing in 4 participants, and a reduction in protrusion in 1 participant (Figure 7A).

On the NMES-applied side, the mean temporal muscle thickness was 4.45 ± 1.24 mm pre-intervention, increasing to 4.78 ± 1.26 mm post-intervention. The degree of temple contour was 0.19 ± 1.19 mm pre-intervention, improving to 0.01 ± 1.28 mm post-intervention. The mean change rates were 7.72% ± 2.09% for temporal muscle thickness and 22.91% ± 17.0% for temple contour. Pre-intervention, the temple contour exhibited depression in 8 participants and protrusion in 6 participants. Post-intervention, the temple contour showed reduced hollowing in 6 participants, enhanced protrusion in 6 participants, and worsened hollowing in 2 participants (Figure 7B).

**Figure 8.**
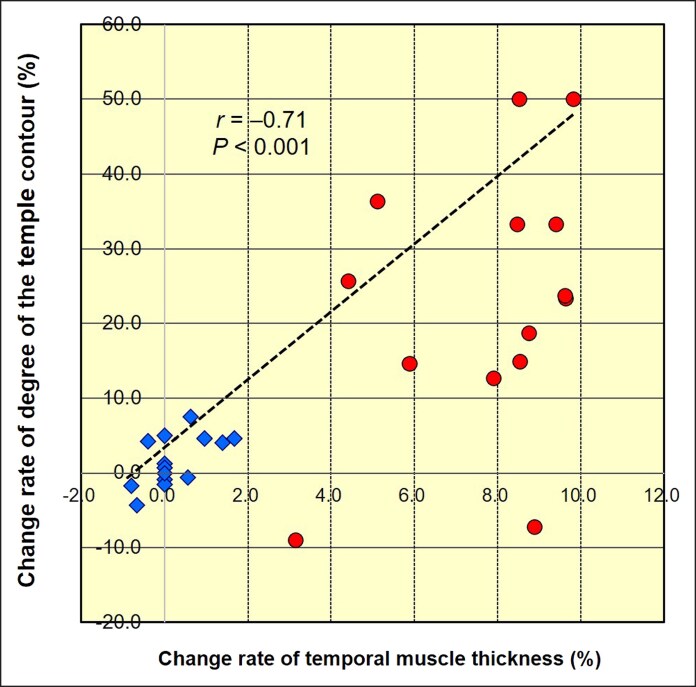
A significantly strong negative correlation was observed between the change rate of temporal muscle thickness and the change rate of temple contour across the 28 temple areas in 14 participants (*r* = −0.71, *P* < .001). Blue diamonds represent the control side, and red circles represent the neuromuscular electrical stimulation (NMES)-applied side. NMES application resulted in an increase in temporal muscle thickness and a corresponding improvement in temple contours, including a reduction in hollowing or enhancement of protrusion.

Statistically significant differences in the change rates of both temporal muscle thickness and temple contour were observed between the control and NMES-applied sides (*P* < .001 for both). Additionally, a strong and significant negative correlation was observed between temporal muscle thickness and temple contour degree across the 28 temple areas analyzed in 14 participants (*r* = −0.71, *P* < .001; Figure 8).

### Subjective Satisfaction Ratings

**Figure 9.**
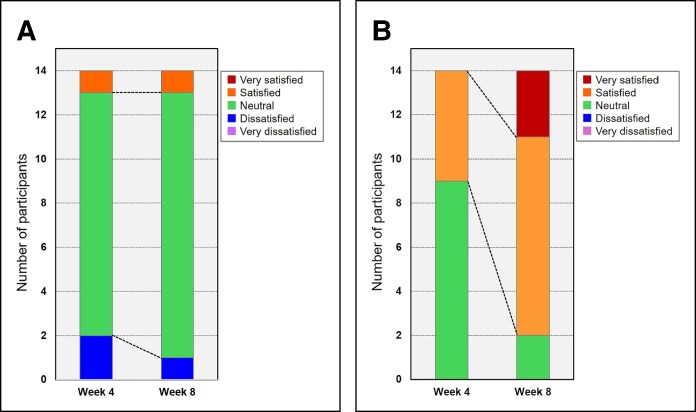
(A) Control side: stacked bar chart shows illustrating the distribution of responses to the 5-point Likert scale questionnaire. The majority of reactions remained “neutral” at both time points, and no statistically significant change was observed between weeks 4 and 8 (*P* = .317). (B) Neuromuscular electrical stimulation (NMES)-applied side: stacked bar chart showing the distribution of responses to the 5-point Likert scale questionnaire. At week 4, most participants selected “neutral” or “satisfied.” By week 8, the distribution shifted markedly toward “satisfied” and “very satisfied,” indicating increased satisfaction following continued NMES use (*P* = .0016).

As shown in Figure 9, to assess participants' subjective impressions, a 5-point Likert scale questionnaire was administered at weeks 4 and 8. The responses for the control side remained relatively unchanged. Most participants rated their impressions as “neutral” at both time points (week 4: *n* = 11, 78.6%; week 8, *n* = 12, 85.7%), and no statistically significant difference was observed (*P* = .317).

In contrast, on the NMES-applied side, satisfaction levels improved markedly over time. At week 4, 64.3% (*n* = 9) of participants responded “neutral,” whereas at week 8, 85.7% (*n* = 12) responded “satisfied” (*n* = 9) or “very satisfied” (*n* = 3). The Wilcoxon signed-rank test revealed a statistically significant improvement in satisfaction from weeks 4 to 8 (*P* = .0016).

### Representative Case Presentation

**Figure 10.**
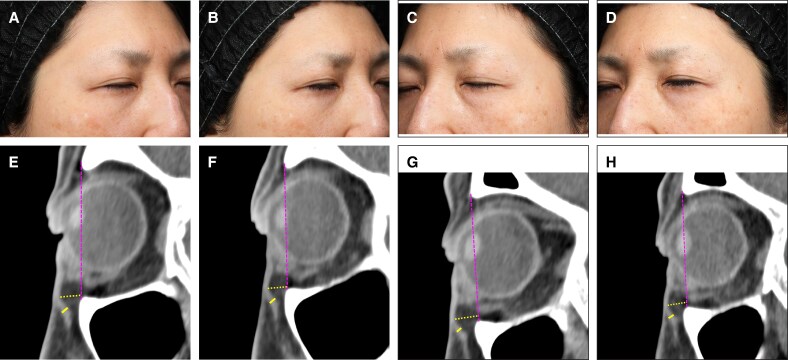
Photographs of the control side at (A) baseline and (B) week 8 in a 54-year-old female participant, showing no perceptible change in the infraorbital region. Photographs of the neuromuscular electrical stimulation (NMES)-applied side (C) at baseline and (D) week 8, showing a visible reduction in the baggy eyelid and attenuation of the nasojugal groove after treatment. (E, F) Sagittal computed tomography (CT) images of the control side at baseline and week 8, respectively. Orbicularis oculi muscle (OOM) thickness (thick yellow line) remained unchanged at 1.47 mm, and the length of orbital fat herniation (thin yellow line) decreased slightly from 5.53 to 5.50 mm (−0.60%). (G, H) Sagittal CT images of the NMES-applied side at baseline and week 8, respectively. OOM thickness increased from 1.40 to 1.60 mm (14.29%), and the length of orbital fat herniation decreased from 6.33 to 5.83 mm (−7.89%).

**Figure 11.**
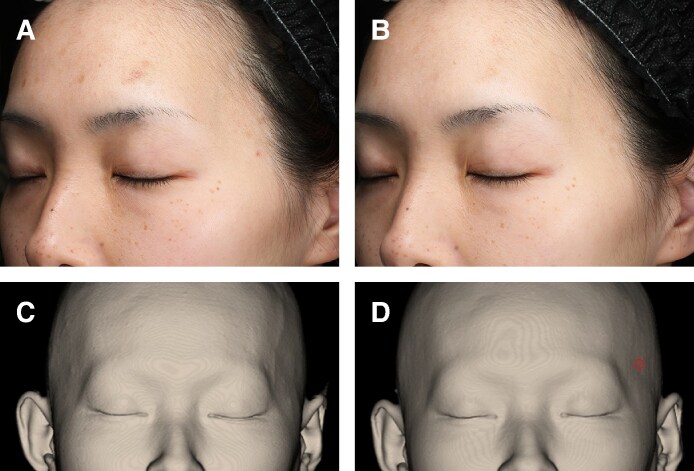
Photographs of the neuromuscular electrical stimulation (NMES)-applied side (A) at baseline and (B) week 8 in a 40-year-old female participant, showing a visible reduction in temporal hollowing after treatment. Hair-removed three-dimensional computed tomography (CT) images of the temporal region (C) at baseline and (D) week 8, respectively. Improved temple contour due to an increase in temporal muscle volume is evident on the NMES-applied side (red circle). Specifically, temporal muscle thickness increased from 5.07 to 5.47 mm (+7.89%), and the depth of temporal hollowing improved from −1.83 to −1.60 mm (−12.73%). No morphological changes were observed on the control side.

Representative cases of the participants treated in this study are presented in Figures 10 and 11.

## DISCUSSION

Age-related changes in the periocular area, including loss of skin elasticity, OOM thinning, and the subsequent anterior prolapse of orbital fat, primarily contribute to baggy eyelids.^11,13,14^ Similarly, temporal hollowing associated with temporal muscle atrophy is a recognized hallmark of facial ageing.^12,15^ These factors contribute to an aged appearance.

In the present study, we objectively assessed the effects of a thin-film NMES electrode device equipped with hydrogel patches designed to fit the infraorbital and temple areas. High-resolution 3-D CT images were used to evaluate morphological changes, enabling a detailed assessment of NMES-induced changes in facial contours and underlying muscular structures. Our results yielded new insights into the impact of NMES on facial morphology.

In the infraorbital area, NMES application significantly increased OOM thickness and reduced the severity of orbital fat herniation. A strong negative correlation between OOM thickness and the length of fat herniation suggests that the NMES-induced increase in OOM thickness may ameliorate periocular laxity. Traditionally, repetitive contractions of facial expression muscles have been implicated in the development of dynamic wrinkles, which typically appear perpendicular to the vector of muscle contraction.^16,17^ This classical dictum is best exemplified by hyperactive muscles such as the frontalis and corrugator supercilii, which generate glabellar and forehead lines. Importantly, other expression muscles, including the nasalis, OOM, and orbicularis oris, also contribute to wrinkle formation. For example, contraction of the lateral portion of the OOM gives rise to crow's feet, whereas the nasalis produces bunny lines at the nasal root, and the orbicularis oris generates perioral vertical lines. In contrast, the present study focused on the OOM and temporal muscles, which are more susceptible to age-related atrophy than to hyperactivity. Thinning of these muscles contributes to structural changes such as orbital fat herniation and temporal hollowing, thereby accentuating an aged appearance.^9,12,14^ Accordingly, the hypertrophic effect of NMES observed in our study is unlikely to exacerbate wrinkle formation; rather, it may restore supportive muscular volume and improve contour. Thus, the relationship between facial muscle activity and skin ageing should be considered dual: overactivity can promote dynamic wrinkle formation, whereas atrophy may worsen static hollowing and fat prolapse. Our findings support the latter perspective by demonstrating that augmenting muscle mass through NMES can ameliorate age-related morphological changes without contradicting the established understanding of dynamic wrinkle formation. In prior studies, evaluations of facial NMES effects have focused mainly on superficial appearance changes^4,6^; however, this study demonstrated that OOM changes play a central role in visible rejuvenation around the eye.

Similarly, NMES application in the temple area significantly increased temporal muscle thickness and improved temple contour by reducing temple hollowing or enhancing temple protrusion. A strong negative correlation between temporal muscle thickness and the change rate in temple contour indicates that NMES-induced muscle hypertrophy may help counteract one of the key anatomical features of facial ageing. Therefore, NMES is a promising noninvasive strategy for addressing age-related structural changes.

No objective thresholds for clinically relevant improvements in orbital fat herniation and temporal hollowing have been previously established. Therefore, in this study, we focused on the objective anatomical relationships between changes in muscle mass and overlying soft-tissue morphology. The present findings showed statistically significant and proportionally greater improvements in NMES-applied areas compared with in controls. Additionally, strong negative correlations observed between muscle thickness and the corresponding contour improvements (OOM vs orbital fat hernia, *r* = −0.73; temporal muscle vs temple contour, *r* = −0.71) were used as evidence of a biologically relevant response. Considering the magnitude of change relative to known age-related decline,^7,9^ the observed effects may be interpreted as clinically meaningful in the context of noninvasive interventions. These associations support the clinical relevance of the observed morphological changes despite the absence of a defined MCID.

Notably, Shay et al^12^ reviewed classification and treatment options for temporal hollowing and highlighted the aesthetic impact of even subtle volumetric differences in this region. Furthermore, Okuda et al^9^ demonstrated that age-related atrophy of the OOM occurs gradually over decades, suggesting that a short-term increase of approximately 13% in OOM thickness could be of clinical relevance.

The efficacy of NMES in promoting muscle hypertrophy has been widely recognized.^1-3,18^ NMES stimulates peripheral nerves transcutaneously, thereby inducing repeated cycles of muscle contraction and relaxation.^19,20^ Consequently, the muscle mass increases. We used a split-face design to compare the changes in the NMES-applied side with those on the control side. Moreover, by incorporating quantitative analysis using 3-D CT, the effects of NMES were evaluated with greater precision and objectivity.

A notable strength of this study lies in the use of detailed high-resolution imaging and measurement techniques, including subcutaneous structures, in contrast to many previous studies that were limited to surface assessments of the face.^4,6^ Thus, the present findings offer scientific and objective evidence regarding the impact of NMES.

The results of this study highlight the potential of facial NMES therapy in novel clinical applications. Specifically, targeted stimulation of the OOM and temporal muscles demonstrated the potential to noninvasively ameliorate age-related morphological changes in the infraorbital and temple areas. This approach may serve as a valuable therapeutic option in the field of antiaging medicine. Historically, interventions for age-related facial changes have largely relied on invasive procedures, including filler injections and surgical procedures.^14,21-23^ Conversely, NMES-mediated muscle augmentation offers a low-risk, home-based alternative that eliminates the need for downtime, thereby representing a significant advancement in aesthetic and rejuvenation therapies.

In addition to the objective improvements in muscle thickness and contour, the present study also demonstrated that participants experienced a significant increase in subjective satisfaction regarding the NMES-applied side. Although the absolute changes in temporal morphology may appear modest (eg, 0.33 mm in the temple), the significant shift in satisfaction ratings—validated by a Likert-scale questionnaire—suggests that even subtle anatomical improvements may be perceived as meaningful by users. These findings suggest that participants perceived a meaningful benefit only on the NMES-applied side, as reflected by the significant improvement in subjective satisfaction scores over the 8 weeks. In contrast, no such improvement was reported for the control side. These findings highlight the importance of incorporating subjective outcome measures when evaluating noninvasive aesthetic interventions, as they may better reflect real-world user experience and perceived effectiveness.

Nevertheless, this study has some limitations. First, it was designed to evaluate the morphological changes in facial structures and did not assess the safety of NMES. Further longitudinal studies are needed to assess the long-term safety of NMES. Second, the study population was limited to healthy middle-aged women. However, the effects of NMES on different degrees of ageing progression and sex differences remain unexplored. Future research including participants of a broader age range and both sexes is warranted. Third, only the short-term intervention effects were evaluated. The long-term sustainability of the observed changes, as well as the potential cumulative or diminishing effects of repeated NMES use, remains unknown. Fourth, this study primarily focused on muscle mass and facial contour. The effectiveness on other tissues, such as the skin, superficial musculoaponeurotic system, and subcutaneous adipose tissue, was not assessed. As these tissues also contribute to age-related facial changes,^7,24^ future studies should include a more comprehensive multi-tissue analysis. Fifth, although the study demonstrated significant morphological improvements with NMES, it was conducted as a pilot investigation without a priori power calculation. The sample size was determined based on practical constraints and the exploratory nature of the study. However, post hoc power analysis using the observed data suggested that the study was adequately powered to detect meaningful effects. Future studies with a larger, prospectively powered cohort are warranted to confirm and expand on these findings. Sixth, this study did not include a qualitative assessment of aesthetic changes, such as standardized photographic evaluations or blinded ratings by observers. Thus, although objective measurements of muscle mass and contour were obtained, it remains unclear whether these changes translate into perceptible improvements in appearance. Seventh, although all radiographic measurements were conducted jointly by 2 experienced radiologists and were averaged over 3 trials, formal assessment of inter- or intra-rater reliability was not performed. Even minor discrepancies in the placement of reference points can influence measurement outcomes, especially when evaluating subtle morphological changes. Future studies should incorporate reliability testing to ensure measurement consistency and reproducibility. Eighth, the questionnaire-based evaluation of participant satisfaction, while informative, may be subject to expectation bias, especially in the absence of a blinded control or sham intervention. Ninth, there is currently no universally accepted threshold to define clinically meaningful changes in orbital fat herniation or temporal hollowing. Consequently, the clinical significance of the observed changes remains hypothetical, albeit supported by strong anatomical correlations. Although no universally accepted MCID has been established for orbital fat herniation or temporal hollowing, we addressed this limitation by quantifying the changes and comparing them with known age-related decline. For instance, the observed NMES-induced increase in OOM thickness averaged approximately 13%, which is comparable in magnitude to the degree of muscle atrophy typically observed over multiple decades of ageing, as reported in previous CT-based studies. Moreover, the strong negative correlations between muscle thickness and morphological changes (OOM vs orbital fat herniation, *r* = −0.73; temporal muscle vs temple contour, *r* = −0.71) further support the biological plausibility of these effects and help mitigate the impact of the absence of established clinical thresholds. Tenth, the study's split-face design, while controlling for interindividual variability, may also introduce inter-side interaction effects that could influence results. Finally, the relatively short follow-up period (8 weeks) precludes any conclusions regarding the durability of the effects or long-term safety of repeated NMES use. Future trials incorporating extended follow-up, larger and more diverse populations, and validated aesthetic outcome measures (eg, Global Aesthetic Improvement Scale, photographic scales) are warranted to establish clinical efficacy.

## CONCLUSIONS

This pilot study demonstrated that facial NMES significantly increased the thickness of the OOM and temporal muscle after 8 weeks of intervention, as assessed by high-resolution 3-D CT imaging. Strong negative correlations between muscle thickness and the corresponding anatomical changes were found. Although these findings indicate that NMES can induce an increase in muscle mass in targeted facial regions, no qualitative or blinded aesthetic assessments were conducted. Therefore, claims regarding aesthetic rejuvenation cannot be substantiated at this stage.
